# Menopause apps: Personal health tracking, empowerment and epistemic injustice

**DOI:** 10.1177/20552076251330782

**Published:** 2025-04-27

**Authors:** Elizabeth Sillence, Alison K Osborne, Emma Kemp, Kerry McKellar

**Affiliations:** 1 Psychology Department, 5995 Northumbria University, Newcastle upon Tyne, UK; 2 School of Psychology, 7315 The University of Sheffield, Sheffield, UK

**Keywords:** Menopause, mhealth, smartphone apps, empowerment, digital health, information, epistemic injustice, personal health tracking, FemTech

## Abstract

**Objective:**

The majority of work in FemTech has focused on menstrual tracking apps but menopause apps are growing in popularity potentially offering greater empowerment for peri and post-menopausal women. Surprisingly little is known about women's actual experiences of using these apps, and how they relate to the epistemic injustice often experienced in relation to menopause. Therefore, the aim of this study is to investigate the role menopause apps play in women's experience of empowerment and epistemic injustice.

**Methods:**

We collected qualitative data, through an online reflection exercise and interviews, from 29 peri and post-menopausal women in the United Kingdom who had experience of using menopause apps.

**Findings:**

The analysis identified two themes (1) Acquiring more knowledge on menopause and (2) Apps as a tool for communication. Women used apps to find out more about menopause symptoms and to track their personal health data. ‘Armed’ with evidence, the apps were used as a tool to combat epistemic injustice often experienced by women in healthcare settings. Women felt empowered by using the apps with an increased sense of confidence and knowledge about their own menopause experience.

**Conclusions:**

Our findings add to the discussions around empowerment in women's health and the potential role of digital technology in supporting knowledge and decision-making. The findings also shine a light on the current debate around the medicalisation of menopause and suggest that understanding how healthcare professionals engage with menopause apps will be important in developing a more holistic picture of FemTech in healthcare.

## Introduction

The menopause is a naturally occurring event for women (We use the term ‘women’ in this paper to refer to anyone assigned female at birth. This follows the general use of the term ‘women’s health’ in the Human Computer Interaction (HCI) community to discuss health issues around menstruation and menopause. The authors acknowledge not all people who experience menopause identify as women. Where possible we have referred to ‘people’ or ‘individuals’ rather than ‘women’ to be as accurate and inclusive as possible but have also used ‘women’ when not to do so would involve inappropriate generalisation.) and is marked by the point at which an individual has gone 12 months without menstruating. Most women experience menopause in their late 40s or early 50s, and it is a heterogeneous experience^1^ with some individuals experiencing few or no symptoms, whereas for others it impacts on the normal functioning and quality of life.^2^ Symptoms of menopause include hot flushes, anxiety, brain fog and migraines and can begin several years prior to menopause, during a period known as perimenopause. The main medicine treatment for menopause and perimenopause symptoms is Hormone Replacement Therapy (HRT). Talking therapies such as Cognitive Behavioural Therapy and lifestyle interventions can also help with physical symptoms such as hot flushes and with low mood and anxiety.^3^ Menopause is socially and culturally constructed^4^ and as such, needs to be understood as integral to life and not a discrete biological event.^5^

Women often report feeling uninformed about menopause and its treatments^6–8^ and this lack of knowledge can affect women's perceptions of their menopause experience.^9^ Poor communication and insufficient support can also leave women feeling isolated during menopause.^10^ The COVID-19 pandemic has accelerated the provision of digital resources for menopause, and it is clear that people are turning to websites, social media^11^ and mobile health apps^12^ for information and support on menopause.

Mobile health applications (mHealth apps) provide a resource across a range of health topics including women's health.^13–15^ Typically, menopause smartphone apps are patient facing health and wellness Digital Health technologies.^16^ Menopause apps vary in quality and functionality,^17^ for example, they may offer information and advice on menopause and treatments or contain multiple tracking features allowing women to track symptoms and periods as well as generating visualisations of tracked data. Some provide access to community forums or to non-interactive peer experiences. Some apps offer more limited features and information^12^ while few contain expert input.^18^ These apps are typically unregulated although some apps, for example, Balance (https://www.balance-menopause.com/balance-app/) have been certified by the Organisation for the Review of Care and Health Apps^19^ who review and approve apps for the NHS and multiple national health bodies around the world. User reviews of menopause apps suggest that individuals feel empowered through their use and gain confidence and knowledge regarding menopause, particularly in relation to the app's self-tracking features.^12^

This is an interesting finding given the critical discourse around FemTech technologies such as period and fertility trackers. Scholars such as Hendl and Jansky^20^ and Epstein et al.^21^ point out issues with the marketing of FemTech including sexist stereotypes and reinforced social inequalities and they question the promise of empowerment for users in relation to reliable knowledge and control of their bodies.

Patient empowerment reflects a power shift away from healthcare professionals (HCPs) towards patients and in the wider context of health promotion is seen as a process through which people gain greater control over decisions and actions affecting their health.^22^ A recent Lancet review series^23^ has proposed an empowerment model approach to menopause in which women become expert in their own condition and act as equal partners managing their care alongside HCPs.

However, reviews of apps often find limited ‘empowering features’. A review of those cancer apps, which stated user empowerment as a key goal, found that few apps promoted a robust conceptualisation of patient empowerment and they varied greatly in terms of quality and functionality.^24^ Menstrual tracking apps often promise empowerment through knowledge and control over your body^25^ despite concerns around their accuracy in predicting ovulation.^26^

Moreover, Hendl and Jansky^20^ analysed the promotional materials of menstrual tracking apps and argued that testimonial injustice was observable in the promotional narratives of these apps, diminishing women's credibility as people who can ‘trust their own experiences and gather reliable knowledge about their own bodies’.

Running through all these arguments is the fundamental issue of knowledge and how personal experience acquires the status of knowledge or evidence in healthcare.^27^ Here, the role of technology remains uncertain. Social spaces on the internet, for example, have the potential to allow people, particularly those with stigmatized or contested illnesses, to make themselves more visible and understandable to others.^27,28^ However, in a healthcare context that prioritises ‘objective’ scientific decision-making this may not go far enough and researchers considering the role of technology in turning experience into knowledge and evidence argue that technologies need to provide other forms of evidence above and beyond personal testimonies.^27^

Personal health tracking may be one way in which such additional forms of evidence could be generated. The collection, storing and monitoring of personally generated health data continues to be seen as a way of encouraging people to engage with their health especially in relation to long term health conditions and reflecting on personal health data may support self-discovery.^29^ However, Campo Woytuk et al.^30^ have also argued that the role of technology is not to act as a gatekeeper to knowledge of the body and the actual design, development and delivery of technologies can be impacted by epistemic injustice,^31^ and the epistemology of apps can involve selective and algorithmic bias.^20^

Epistemic injustice takes place when knowledge contributions of an individual or community are afforded less visibility due to direct or indirect discrimination or put more simply ‘when someone is wronged in their capacity as knower’.^32^ Testimonial injustice is one form of epistemic injustice where the speaker is assigned an unfair deficit of credibility owing to a prejudice on the hearer's part. Patients or people seeking healthcare are often vulnerable to testimonial injustice as their emotional and or cognitive characteristics often downgrade the credibility of their testimonies.^33^ Patients can experience epistemic injustice in different ways. At the most basic level they can simply be ignored or not acknowledged, or HCPs may listen to patients but then fail to see the value in their testimonies, judging them unimportant, irrelevant or poorly articulated. Certain types of patients, for example, those with mental health difficulties may encounter challenges in being listened to or taken seriously as an epistemic resource.^27^ Epistemic injustice is particularly noticeable in contested illnesses such as chronic fatigue syndrome, fibromyalgia and recently long covid.^34–36^

In women's healthcare the problem may be compounded by women's structural marginalisation.^37^ Fricker^32^ suggests HCPs rarely adopt these negative positions on purpose but nevertheless they present a substantial barrier to women seeking to access support and treatment for a range of health issues including menopause.

Mindful of these tensions, we focus on menopause apps and respond to Hendl and Jansky's^20^ call for further research into users’ actual experiences of epistemic injustice and their experiences of using FemTech. They note that their observations on epistemic injustice in the discourse of apps relate to the promotional narratives told by the apps and do not necessarily reflect personal usage experiences. Studies focusing on women's experiences of app use are emerging in this space. Levy and Romo-Avilés^15^ and Epstein et al.,^21^ for example, interviewed women about their use of menstrual tracking apps and de Boer et al.^38^ examined menopausal women's experiences of self-tracking in general although this study did not specifically examine women's use of menopause apps. The majority of work in FemTech has focused on menstrual tracking apps, less attention has been afforded to menopause apps despite their rising popularity.^39^

While the word ‘empowering’ was frequently mentioned in user reviews of menopause apps^12^ we do not know how these individuals experienced empowerment and whether the apps perpetuate or alleviate epistemic injustice in relation to menopause. Given the current focus on empowerment within menopause^23^ we take this opportunity to draw on the concepts of empowerment and epistemic injustice as a way of exploring the role of menopause apps in women's experiences of menopause. We present data from 29 women detailing their use of menopause apps and ask: What role do menopause apps play in women's experience of empowerment and epistemic injustice?

## Method

Following approval from the authors’ institutional ethics committee [REF:362], women were recruited to take part in a multi-method qualitative study to understand the role of menopause apps. Recruitment took place during 2023 via social media, physical posters and organisations including Menopause café. Women were eligible to part if they had any experience of using a menopause app, either currently or in the past.

### Sample characteristics

Twenty-nine participants, all of whom identified as women, aged between 42 and 57 (mean 50.9 years), all from the United Kingdom consented to take part in an online reflection exercise. Women were invited to leave their email addresses if they wished to take part in a follow up interview. Fourteen women were therefore contacted and invited to take part in a follow-up interview and 10 participants aged between 48 and 57 (mean 52.1 years) agreed to take part. The majority of participants were taking HRT at the time of the study and were using a menopause app and had seen a GP regarding menopause (see Table 1 for participant information and Table 2 for details of the apps used).

**Table 1.** Participant information (*N* = 29).

	Reflection exercise	Interviews
Sample size	29	10
Age		
Range	42–57 years	48–57 years
Mean (SD)	50.9 years (3.80)	52.1 years (3.45)
HRT		
Currently taking HRT	22	10
Not taking HRT	7	0
Menopause app use		
Currently using app	14	6
Previously used app	15	4
Accessed a GP for menopause		
Seen a GP	27	10
Not seen a GP	2	0
Menopause apps used		
Balance	22	10
Caria	1	0
Health & Her	1	0
Menopause matters*	2	0
Could not recall	3	0

*Menopause matters forum was misidentified as an app, rather than an online forum by two participants completing the online reflection exercise. However, their data referred to features of app use and therefore we included their data and left their ‘named app’ unchanged for transparency. We have not included Menopause matters in Table 2 as that provides an overview of apps and their features.

**Table 2.** Overview of apps used.

	Balance	Caria	Health & Her
Self-tracking features	X	X	X
Generates health report	X		
Peer support	X	X	
Content and advice from experts	X	X	X
Provider type	Founded by GP and menopause specialist	Developed with leading experts in women's health	GP reviewed
ORCHA certified*	X		X

*The organisation for the review of care and health apps.

### Reflection exercise

All participants completed a reflection exercise online consisting of eight prompts designed to encourage reflection on their experiences of using menopause apps [see supplementary material]. These prompts asked them to consider their motivations for downloading the app, their initial and longer-term interactions with the app and to reflect on the role the app played, if any, in their menopause. Participants were encouraged to write freely in response to the open-ended prompts developed on Qualtrics software.

### Interviews

Ten participants took part in a follow-up individual interview to explore some of the experiences from the reflection exercises in more depth. The interviews began by asking participants to describe their menopause journey so far and think about the sources of information and support they had used. The schedule then focused on menopause apps more specifically and asked participants to think about how and why they had first downloaded an app and then to reflect on how they had used apps over time. Participants were prompted to discuss the features of the app that they used or did not use and why and to note any changing patterns of use over time. The interviews then moved onto the perceived outcomes of the app use and here participants were prompted to consider how using the app had made them feel, any changes that had occurred through using the app and the if not already covered the notion of empowerment, for example, *it has been suggested that apps can be empowering, how do you feel that statement relates to your own experience using menopause apps?* [see supplementary material for interview guide]. Individual interviews took place via Teams or telephone and lasted on average 30 min; all interviews were all conducted by the first author. Interviews were audio recorded and either automatically transcribed and checked or were transcribed by hand.

## Analysis approach

Responses to the reflection exercises were compiled and printed into paper format. Interviews were transcribed and printed. Following Braun and Clarke^40^ six phases, the qualitative data from the interviews and the reflection exercise were analysed together through Reflexive Thematic Analysis. Three authors (ES, AO, EK) read and reread both data sets to familiarise themselves with the data to develop preliminary notes and codes focusing on how women described their use of apps and their relationship with their experience of menopause. Three of the authors then met for two, 2-h roundtable sessions to consider codes and potential themes and to confirm data saturation had been reached. The first author then coded, grouped and developed themes for further discussion and refining using sematic, or surface-level, readings to consider the participants own words about their experiences with menopause apps and latent readings to look for the unspoken context of app use. The completed set of draft themes was then reviewed by KM. Feedback and revisions were made to the themes, and the final set of themes and sub themes were then named and presented below.

*Reflexivity statement:* All four authors identify as women and two were familiar with menopause apps while two had not encountered them before. The first author carried out all the interviews and was familiar with using menopause apps and this allowed for greater relevance and depth in the interviews. However, she made a conscious effort to remain aware of any biases she held in relation to her knowledge and experience. We engaged as a team regularly throughout all stages of the study to ensure our diverse perspectives were considered and to allow challenges to any preconceptions.

## Findings

In response to the research question: What role do menopause apps play in women's experience of empowerment and epistemic injustice we present two themes: (1) Acquiring more knowledge on menopause and (2) Apps as a tool for communication (see Table 3 for an overview of themes and subthemes).

**Table 3.** Overview of themes and subthemes.

Theme	Subtheme
Acquiring more knowledge on menopause	Figuring it out myself
	Normalising the experience
	Taking control and deciding on next steps
Apps as a tool for communication	Figuring it out myself
	Normalising the experience
	Taking control and deciding on next steps

## Acquiring more knowledge on menopause

The first theme illustrates how women were able to use the app to gain a comprehensive picture of their unique menopause experience. This involved reading about symptoms linked to menopause and linking their experiences to ‘normal’ issues supported by the sense that they were ‘not alone’. Women described how the apps helped visualise the patterns associated with their menopause as well as providing reassurance and information which facilitated perspective taking and decision-making around next steps.

### Figuring it out myself

Many women described using the app to help them make sense of their situation. This often involved using the tracking element of the app to record and monitor symptoms. This allowed women to gauge the severity and the longevity of their symptoms. Importantly here, the app did not reveal new information to them about their own bodies but helped women to make sense of their symptoms, to connect symptoms and see patterns emerging. This sense of joining the dots ‘being able to log symptoms and see patterns emerging’ [P24] was prevalent across women and was sometimes described as carrying out ‘detective work’[P2].

Being able to see all symptoms logged in one place within the app was referred to as a ‘lightbulb moment’ [P1] or as one participant put it ‘I'd seen this list of symptoms and realised that was me’ [P24]. The app provided women with a sense of the scope and scale of their symptoms and thus an immediate and more comprehensive picture of their menopause experience.

It made me realise why I was feeling the way I did….and a better understanding of my symptoms [P2].

It's through using the app and my tracking that I’d kind of got to realise what those patterns were and then actually thinking it can’t just be I’m getting old and why should I have to suffer [P5].

For many women this was the first time they realised that some of the symptoms they were experiencing were menopause related rather than standalone as P23 explains:

So many of my symptoms were listed in the app which I was asked to use to log my experiences each day. I didn't even realise until then just how much of what I was experiencing was down to menopause. Menopause is no longer a mysterious dark art, it has tangible, recognised symptoms which the app demonstrated. [P23]

### Normalising the experience

Realising that so many symptoms were linked to their menopause was an important moment for many women and one that helped to normalise their experiences. Here, many women described how apps provided reassurance and were a ‘place to go when feeling uncertain about what is happening’[P8] or that they ‘help *me* know what's going on’ [P7]. Women felt that apps helped them recognise that symptoms were explainable, there was an underlying reason for their symptoms, and this afforded women the reassurance of normality. Symptoms were experienced by others going through the menopause and were normal. Here, many women contrasted this feeling of normality with previous feelings of ‘going mad’ [P17] [P23] [P19] or ‘crazy’ [P3]. As P24 explains below:

Suddenly I realised symptoms I'd dismissed or got used to were also menopause and I wasn't going mad! [P24]

Part of normalising the experience came through a sense of ‘not being alone’. The community features on the apps allowed women to read about other people's experiences and thus provided a sense of comfort:

I don't feel so alone - it's good to know that others are feeling the same and experiencing the same. [P14]

It was very helpful to glean information and insights from the other experiences. It made me feel slightly better informed. It was good to see I was not alone in my horrible experiences. [P4]

### Taking control and deciding on next steps

Using the apps helped to build a clearer picture of the menopause journey and allowed some women to seek coping strategies and think about treatment options. Some of the women talked about finding perspective in relation to menopause and taking control of their lives. It was not necessarily about the app providing solutions but using the app to understand what was happening and why. For these women, the information gained from the apps allowed them a better sense of control over this stage of their lives and where appropriate to decide on next steps. For many women the apps enabled a sense of empowerment of their menopause experience.

It helps me to be prepared in menopause and get on with my life as much as possible. [P5]

I felt empowered if that's the word for the information I was able to gain and just feeling like I wasn’t alone and feeling like I could cope with it that I had to do the best that I could and that was the thing that gave me that sense of control I think over ok this is a stage in your life ok this is fine. [P19]

For others, using the app was just the start of ongoing information seeking and reflection. The app generated further questions to be answered around how best to help yourself.

OK, I think this is me, this is what's happening to me. This explains a lot. Thank the Lord. There is an explanation, but I need to know a lot more because I want to if I'm going to sort of pursue a path of trying to get help I just need to have like as many facts as I can. [P1]

I did look before I before I chose that [app] and a lot of them were trying to solve the problems. They'll give you answers, and I don't necessarily think an app can do that. I think you need to understand what's happening to you first before you go and decide which answers are gonna be right for you. [P26]

A number of women reported that the information gained through the app helped answer specific questions that informed decision-making, for example, around seeking certain treatment options. They were also able to refine questions and tested them through their personal health tracking, whether by checking specific symptom patterns or the efficacy of supplements.

I like the tracking but more the reviewing element so you can look back and see whether you really are having more headaches. [P12]

I wanted to look back after a month on the supplements to see if it had reduced my symptoms. [P28]

However, not all apps were seen as useful and sometimes their use led to over thinking about menopause symptoms.

I found myself I kept going back to the app and thinking, oh, what about this? What about that? And it was becoming more of my day and I thought that's not that's not a helpful behaviour really. [P25]

## Apps as a tool for communication

The second theme captures the way in which women used the menopause apps to push back against the perceived dismissal and difficulties they reported in accessing the information and support they sought regarding menopause and importantly around access to HRT. Discussion of epistemic injustice, although not a term used directly by participants, was strongly felt by many in their encounters with HCPs. The theme also highlights the functionality of the app and its role in facilitating conversations with HCPs.

### Armed with evidence

Many women spoke of the difficult times they had trying to get support from their GP regarding menopause. This included recognition they were going through menopause or had problematic perimenopause symptoms, refuting alternative diagnoses such as depression and in particular around access to HRT. Women often reported feeling vulnerable and were looking for advice and support from their GPs. This resulted in frustrating conversations and feelings of ‘being fobbed off’ [P1] or not listened to.

I went to the GP just like feeling a bit low, periods slightly out and things like that. So I thought I'll make a well woman appointment and it was horrendous. And I was like, I don't know if this is menopause or what it is if it's not, then obviously we'll have to look at something else. So yeah, she dismissed me, said that it wasn't menopausal stuff. Maybe I was pregnant. I wasn't pregnant. Yeah. If you're not having period, maybe you're pregnant. No, I am having a period, it's just changing. So that wasn't great. [P25]

I know GPs have a lot of a lot of work and things, but they don't seem to like to listen, you know, and you, you turn around and you physically tell them I know my own body. They're just not interested. ….. I was trying to prove to the doctor that I’m going through the menopause cause they are like just No you’re not. [ P21]

Here, many women described a protracted timeline which involved seeing different HCPs, in different locations and clinics, over a number of visits, spanning months and sometimes years. Many women felt they were not listened to, and those willing to keep trying with HCPs evoked battle imagery as they described their preparations. Women talked about being ‘armed with knowledge’ [P14] ‘arm myself’ [P26] ‘wanted to be armed with info’ [P9] ‘go head-to-head’ [P1] ‘So I'm going to fight for it this time’ [P21].

It's empowering to educate yourself and go armed with knowledge when you speak to GPs etc [P14]

Women explained how the app had proved key in terms of providing ‘proof’ [P21] [P1] about their periods or their symptoms, severity and duration and that if it was not for the app giving P8 the information ‘I probably would have given up and suffered in silence [P16].

The evidence was available to women on the reports generated by the app and they took that evidence along with consultations or were ready to show GPs that evidence or had reflected on the evidence before seeing a HCP. Here, P21, P24 and P2 explain:

I think it's been valuable in being able to track my periods, so I effectively had proof for the doctors ‘cause you know they're reluctant to take your word for it unless you can show them something. [P21]

[I said] I've been to the GP and got poo pooed. I've been about these itchy legs and got poo pooed. I've got told it was to do with my blood pressure. I went and saw another nurse and she the doctor and she ripped up the prescription in front of me. I'm desperate. I've now been into this app and this is what I've discovered and I think I need it. [P24]

I know that GPs hate you using google but I said you know I’ve just been onto this app and I can relate to some of these symptoms so I don’t want to be on antidepressants I want to be on something else so I just need to know my options and then he referred me to the nurse. [P2]

Even for the women who had a more positive experience with the GP they still anticipated difficulties and P26, for example, describes being prepared for a battle and how the app was something they held in reserve should they need it in a consultation:

I didn't go in and say right, this is what I want because I might have done had he not had he taken a different approach and said oh you’re fine or you just take an antidepressant or whatever. Then I would have. I would have hit back with well, I've been doing this I've been doing this. But I didn't need to do that. [P26]

### An accessible tool

The app provided an accessible format for personal health data for participants and in a few cases for HCPs. The app allowed participants to record their data in one dedicated place and this conferred advantages over other devices or formats that women had previously used for tracking their health. Some women talked about the difficulty of having to try and remember dates and symptoms while others had previously used fitness trackers or even created their own spreadsheets to record symptoms. These other formats had disadvantages in comparison to the apps as P21 and P7 describe below when detailing the use of the calendar function or a self-developed spreadsheet.

I tried to log in my calendar and like show them that and they're like, Oh no. Because it wasn't like easy to show them because I had to scroll through month and month, but with the app you can just log on period tracker and it showed you the information that it was needed. [P21]

I would see a pattern arising then that's when I downloaded the app and put that in cos I figured it would be easier to carry around an app than it would be to carry around my laptop. [P7]

Participants reported finding the app easy to use, allowing them to ‘log everything’ [P7] and ‘anything’ [P16] and to ‘log symptoms in order of severity’ [P28].

The clarity and single purpose of the tool made women feel confident in being able to show the HCP the ‘right’ information and having an accessible tool to hand was useful in providing HCPs with the information quickly in order to prevent a delay to any treatment.

I can log my periods and symptoms easily which is important now for when I speak to my GP about anything as the first thing they always ask is why don’t you keep a calendar of things then we can see if there's a pattern - which would just prolong any actual help. This app means I already have a history of such things to show. [P26].

Some women found having the report to hand on the smartphone was an important part of the consultations while others described the benefits of printing off the record or looking at the report prior to their consultation.

Further benefits were reported when the GP was also familiar with the format of the reporting. One participant, whose GP suggested she use the app to track her symptoms, reflected on the ease with which they were able to communicate about her symptoms and discuss treatment:

I did it all and then downloaded the report and it from memory it looked almost like a sort of chart thing, but I think because she was so familiar she could look. She could see all the things I'd plotted on there. And it's like, oh, yeah, HRT, it's absolutely going to be a win for you. [P23]

### Facilitating the conversation with HCPs

In addition to the physical presence of the app itself, women felt their use of the app meant they were now in a much better place to tell their story and to present a clear and concise account of their menopause experience or ‘facilitate the conversation’ [P2]. This involved being able to verbalise what they had done so far, what they wanted to discuss going forward and importantly being able to make use of the ‘right language’ for the consulting room.

P1 explained that the app had helped them pinpoint the correct terminology and wondered whether accessing this information earlier might have made her interactions with HCPs more straightforward.

I think some of the terminology is a little bit tricky when you're sort of coming to it new. When I'd seen a GP previously and asked for bio identical and they said we don't do those on the NHS. Then I found that consequently that I should have been asking for body identical. And then maybe I could have had prescription and I've wasted two years. There's things like that just understanding exactly how it would work. [P1]

For others, it was being able to have the confidence or feeling ‘empowered’ [P11, P2] to be able to articulate with fluency what was happening to them and thus pre-empting or anticipating the GPs questions and prompts. They had the confidence to ask questions and to ask for HRT if that was their decision. The role of the app in terms of confidence was explained by P24 below:

It's just been life changing and I would put the app in that because that's what gave me the confidence to go in the way I did to see the menopause specialist… whilst my HRT nurse has been amazing. I would say it's the app that gave me the confidence to do that and it is the app I consider that made that biggest difference in my life. [P24]

Women felt able to articulate an account of symptoms and the actions they had already taken to minimise symptoms. They were able to summarise specific patterns and triggers and to have reflected on alternatives explanations and options. Given the short period of time available in the consulting room, this succinct account was important in conveying the information they hoped the HCP would see as relevant.

I took all that information to the nurse. Totally different experience with her….told her everything that I was experiencing, everything that I've been doing to try and help through supplementation, diet changes and lifestyle changes and things. And she was like, you're doing everything that you can and I think really HRT would be the next step for you. … [P25]

Finally, using the app had primed women to think about answers to a range of menopause related questions. In the HCP meeting, the app served as a mental checklist against which to check the quality and thoroughness of the HCP's assessment.

I wanted to see whether it matched what I was thinking … [the HCP] was very good, in depth and went through similar forms that I'd already been through on the app and came out at the same conclusion as me. [26]

## Discussion

This study aimed to understand the experiences of women using menopause apps and to examine their role in supporting menopause. This is the first study, to our knowledge to focus on women's actual experiences of using menopause apps. Overall, the apps played a positive, supportive role in menopause. Most women used the Balance app which allowed them to find out more about menopause symptoms and to track their personal health data. Most apps were useful in recognising patterns of symptoms and charting menopause experiences. This provided a starting point for exploring coping strategies and treatment options. For a significant number of women, the tracking and data visualisations proved a useful tool in discussions with HCPs in terms of corroborating their menopause accounts or asking for HRT. Women felt empowered by using the apps with an increased sense of confidence and knowledge about their own menopause experience.

Many of the women in our study felt that the menopause app had been an instrumental tool in combatting the epistemic injustice they experienced with HCPs. Having the app to hand or checking it before going into the consulting room allowed women to present a record of their symptoms and concerns in a way that appeared to overcome many of the barriers they felt had been in place beforehand. These findings resonate with the work of Grimme et al.^41^ who found that women felt that collecting data can help legitimise their symptoms during medical appointments. In stark contrast to critics such as Hendl and Jansky,^20^ who analysed promotional materials and cautioned against the narrative that only apps generate self-knowledge and are thus empowering, our study finds that women were already well aware of their menopause symptoms and that their experience of using the apps was subtly different to the one implied in marketing endorsements of FemTech. Women described an active use of the technology in relation to HCPs in which the knowledge they already possessed about their bodies was captured and documented in a format that made it easier for them to collate and present to HCPs. As Mazanderani et al.^27^ note technology may help patients be taken seriously in a context in which objective scientific decision-making is the norm. Using a menopause app did not offer our participants empowerment per se but could be viewed as part of an empowerment process^42^ in which women felt more in control of their menopause and more confident to enter into shared decision-making with HCPs. Viewing empowerment in this way and recognising the role of HCPs necessitates a better understanding of how HCPs engage with menopause apps before we can have a holistic picture of the role of FemTech in healthcare. This is essential given that clinician distrust of patient-generated data remains a barrier to sharing in some contexts.^43,44^

The idea that technology is seen as being helpful in combatting epistemic injustice is encouraging for the design of digital health services and contrasts with technology's role as perpetuating or enabling epistemic injustice in other contexts (see e.g. the work on digital harms.^45^ ). In a healthcare context, thinking about how technology could be better designed to support people going through menopause requires active consideration of epistemic injustice and thinking about how social power and intersectionality affect how and whether knowledge is received.^46^

Women would still like to see a more personalised approach to menopause apps, being able to capture the data that is meaningful for them presented in a way that is meaningful for them^12^ but apps are a useful tool to aid communication with HCPs. Our findings provide an interesting contrast to de Boer et al.^38^ who found that women stopped or reduced their self-tracking habits in menopause because they felt that knowledge was already available to them, or it could not affect change. We note, however, that participants in de Boer et al.'s^38^ study were referring to a wide variety of self-tracking apps include fitness and calorie counting apps rather than menopause specific apps that offer more symptom tracking features as well as information and peer support. Furthermore, some participants had not used any form of self-tracking app and none of the participants were taking HRT. Overall, our findings support the notion that menopause specific apps can play a positive role in menopause for example through symptom reduction^47^ and expand on Sillence et al.'s^12^ study of user reviews in which individuals reported feeling empowered to speak to their HCPs after using menopause apps.

In addition to having the ‘evidence’ from the app, participants reported feeling more confident about discussing their menopause symptoms with HCPs. While data visualisations are perhaps more congruent for a health context, having a more comprehensive picture of their experience was also important in terms of being able to talk about their symptoms, feelings, actions and desires. Ziebland and Wyke^48^ suggest that one of the ways in which online patient experiences impacts health is being able to learn to tell your own story. Participants in our study felt more confident discussing their menopause experiences after using the app. The apps provided access to other people's stories, to information and terminology which all helped women feel more comfortable and familiar with menopause and treatment options, and this allowed our participants to be able to tell their own story for themselves, friends, family and work colleagues and HCPs. In the time-pressured consulting room, anticipating questions, having answers ready and being familiar with the terminology are important features of a successful interaction although this additional work still puts the onus on the patient rather than on the HCP to recognise and be flexible to different presentations and accounts of health and well-being.^33^ Technology has the potential to promote health literacy,^49^ and menopause apps might contribute to a broader health literacy about women's health, especially in situations where education and learning gaps exist for HCPs around menopause.^50^

Our findings on personal health tracking add to the current debate around the medicalisation of menopause. While management of symptoms is important, Hickey et al.^23^ argue that the disease-based model approach to menopause focuses only on the negatives and can lead to women feeling disempowered and over-treated. Furthermore, it has been argued that self-tracking technologies can be seen as promoting an overly medicalised approach to a natural process^51^ focusing on numbers over physical sensations, and Homewood^52^ has cautioned against the design of unnecessary technologies around menopause. However, while many of our participants described their use of the app in relation to seeking out HRT, they also spoke about normalising menopause within the context of their daily lives. Empowerment extended beyond simply accessing HRT but to taking control of their own menopause experience. Personal health tracking with the app was often part of a wider piece of self-care in which participants engaged in increased self-reflection and positive decision-making about their health and well-being. Participants took part in hypothesis testing,^53^ for example, evaluating the effect of supplements on their symptoms and data from the apps was often used personally for reflection rather than for sharing.^54,55^ While the apps provided some opportunities for self-reflection they could be better designed to support empowering experiences, for example, by providing opportunities for meaningful self-reflection rather than interpreting only preset categories and experiences defined by the app.^12,56^ Overall, using the apps had led to positive acceptance around menopause, increased discussion with friends, family and work colleagues, as well as exploration of diet, exercise, supplements and other forms of self-care. Raising the profile of menopause was seen as a good thing and women were positive about their ability to discuss their experiences in different contexts. The findings of this study resonate with a number of the factors identified on the empowerment model for managing menopause^23^ including access to appropriate tools supporting decision making about treatments and access to realistic and balanced information. Ultimately, women become expert in their own menopause experience. Hickey, et al.^23^ stress the need for supportive and informed clinicians and our findings suggest that menopause apps could be a tool that underpins discussions with HCPs and thus supports empowerment in the management of menopause.

It is important to consider the limitations of this study. While all participants were based in the United Kingdom, we did not collect any further characteristics which would have helped to clarify the representativeness of the sample. Understanding how socio-economic status, education, income, overall health status, sexual orientation, race and ethnicity play a role in people's experiences of using menopause apps especially in relation to epistemic injustice is an important piece of future research. Although all our participants identified as women, not everyone experiencing menopause will identify as a woman and research on menopause in gender diverse individuals is limited,^57^ while the use of digital technology for menopause is often predicated on whether users feel the technology represents them.^58^ We also acknowledge that the majority of participants had experience of using the same UK-based menopause app. Whether other menopause apps would play the same role in providing information, contributing to empowerment and combating epistemic injustice for women who use them requires further investigation. Although apps were seen in a positive light by the majority of women who took part in the study not all women were still using an app and so the longer-term role of apps in supporting menopause experience remains unknown.

## Conclusion

Menopause apps provide a valuable role for women, particularly regarding knowledge acquisition and facilitating communication with HCPs. The information gained through menopause apps confirmed and enhanced their existing knowledge of menopause and their symptoms. This increased women's confidence in their knowledge of menopause and in their ability to articulate and discuss their experience and symptoms with HCPs. ‘Armed’ with this information, the apps were able to be used as a tool to combat epistemic injustice often experienced by women in healthcare settings. The women felt they were better listened to, taken seriously, and able to collaborate in decision-making around their health and treatment outcomes having used and engaged with the menopause apps. Understanding how HCPs engage with menopause apps will be important to capturing a more holistic picture of the role of the FemTech in healthcare.

## Supplemental Material

sj-docx-1-dhj-10.1177_20552076251330782 - Supplemental material for Menopause apps: Personal health tracking, empowerment and epistemic injustice Supplemental material, sj-docx-1-dhj-10.1177_20552076251330782 for Menopause apps: Personal health tracking, empowerment and epistemic injustice by Elizabeth Sillence, Alison K Osborne, Emma Kemp and Kerry McKellar in DIGITAL HEALTH
