# Supplementary material for: Menopause apps: Personal health tracking, empowerment and epistemic injustice
Source: Digit Health. 2025 Apr 27;11:20552076251330782. doi: 10.1177/20552076251330782 (PMC12034958; doi:10.1177/20552076251330782)
Supplement: sj-docx-1-dhj-10.1177_20552076251330782 - Supplemental material for Menopause apps: Personal health tracking, empowerment and epistemic injustice [file sj-docx-1-dhj-10.1177_20552076251330782.docx]

**Menopause reflection exercise**

**Please indicate which of the following statement applies to you:**

- I am experiencing menopausal symptoms
- I am experiencing menopausal symptoms and I have NOT had a period in the last 12 months
- I have NOT had a period in the last 12 months and I am no longer experiencing menopausal symptoms

**Which of the following statements best describes you:**

- I currently use a menopause app
- I have previously used a menopause app but I am not currently doing so

**Please provide the name of the app you use/have used. If you use more than one app please name the one you use the most often/have used the most often.**

App name :…………………………………………………………

**Thinking about the app you have named above please answer the following questions. We are interested in your experiences and how you felt using the app so please provide as much detail and information as possible. Thank you.**

- Can you tell us what you were thinking and experiencing when you decided to download the menopause app?
- Please tell us about your first experience of using the menopause app? How did it make you feel? What made you continue to use it?
- What do you like best about using the menopause app? Why?
- What don’t you like about the menopause app? Is there anything you would like to change or improve?
- To what extent do you feel the menopause app has supported you in your menopause and why?
- Some app users have said that menopause apps are *empowering*, can you relate to this statement? Do you agree with the statement and if so can you explain why this is the case?
- We are interested to know whether you would recommend the menopause app you are using/have used to a friend. Could you tell us what you would say (and why) to a friend who asked for your advice about whether to use the menopause app?
- Compared to other apps on your phone, how often to do use your menopause app? Why is this?

**Demographics**

**How old are you?.....................................**

**What is your gender?**

Woman

Man

Trans man

Trans woman

Non-binary

Prefer not to say

Self-identify (please describe…………………………….)

**Are you currently using any treatments to improve your menopause symptoms? (please tick all that apply)**

- - HRT
  - Natural Remedies
  - Other (e.g., Yoga)

**Thinking about when you first started to experience symptoms of perimenopause/menopause, please indicate your agreement with the following statements**

- I felt sufficiently knowledgeable about menopause
- I felt prepared to go through menopause
- I felt comfortable talking to friends and family about menopause
- I felt supported in terms of my menopause
- I sought medical advice from my GP about menopause

If you are interested in taking part in a follow up interview to explore some of these issues in more detail, please leave your email address here and we will contact you.

Email address:……………………………………………………………………

**Follow up Interview guide**

- Motivations for using an app
  - Thinking about why you started using the app – what prompted you to go looking for a menopause specific app /or was it this app in particular?
  - How did this fit into your menopause journey?
- Exploring the use of the different features
  - Can I ask you about the features of the app that you use the most and why?
  - Prompt: Logging, information/articles, community support?
  - Do you use all of them– are you single minded in how you use it – exploratory, what’s your style?
- Using the app and outcomes
  - Can you tell me how you use/have used the app?
  - What has using the app meant to you, what have you got out of using it? It has been valuable? Was it what you expected?
  - Explore: How have you used the tracking feature (if applicable)?
  - Explore: How have you used the information and advice (if applicable)?
  - Explore: How have you used the community or peer aspects of the app (if applicable)
- Usage patterns
  - Are you still using the app? Why or why not what made you stop? Are you getting the same support/information elsewhere/still tracking etc?
  - When are the apps most useful? If you’ve stopped for now, would you return to using it?
- Trust, privacy, security perceptions of the app?
  - To what extent do you think about privacy and security when using the app?
  - To what extent do you trust the App developer/source of information.
  - Was the app free to use? Does that make a difference?
- Apps in relation to other digital and non-digital forms of support
  - Have you used other digital resources in relation to the menopause
  - Have you visited the GP, spoken to friends and family about menopause (before or after using the app?)
  - How do these different sources of advice/support compare?
